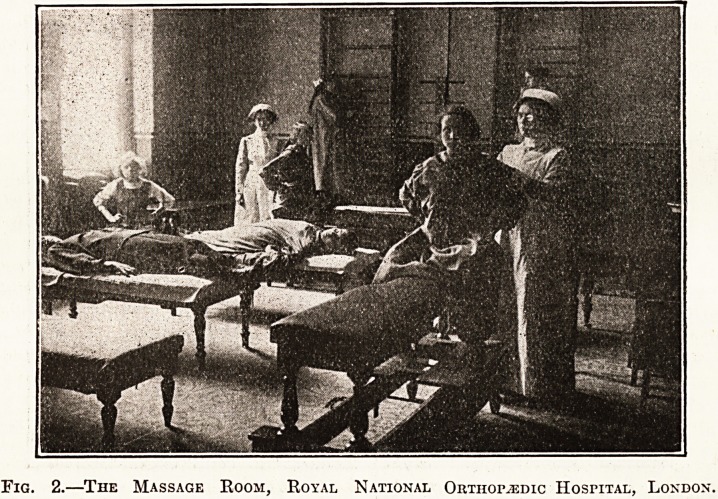# The Modern Orthopædic Unit

**Published:** 1914-01-24

**Authors:** 


### THE ORTHOPÆDIC DEPARTMENT.*

### V.—The Modern Orthopædic Unit (*continued*).

### THE TREATMENT DIVISION.

We now come to what is, after all, one of the most important, if not the most important, divisions of the department—the treatment division. In-patient treatment is, of course, given in the ward and the operating room attached to it; minor treatment and such treatment as is of an operative nature to be administered in the out-patient department is given in the division we have dealt with in the preceding article. But there remain a large number of cases in which the patients are not suitable either for ward or for out-patient treatment in the ordinary sense of the term. We allude to cases that require electrical and massage treatment, those that need educative treatment, those that want instrumental treatment, and those that need exercises and constant supervision by trained assistants. For these a separate division must be created.

It is perhaps well to dilate somewhat on the necessity for such a separate treatment department, since the necessity for it is not at present so well recognised as it should be. A large part of the orthopædists' work lies in such a department. One of the truisms of the science of orthopædics is that the surgeon should hold his hand as far as possible, and that he should exhaust all other means before he resorts to operation. It is well to bear this in mind. Modern orthopædy succeeds with manipulative methods—methods that demand on the part of the surgeon and his assistants infinite care and patience, on the part of the patient infinite courage and equal patience. A case of club-foot in a young child, for instance, is best treated by careful and continual manipulation; such manipulation, in the vast majority of cases where no gross structural defect exists succeeds in overcoming the deformity, whereas operation, unless it is followed up by equally careful after-treatment, very often fails in achieving that desired result.

The treatment division, in essentials, should comprise a plaster room and instrument room with workshop attached, an exercise room large enough for crawl exercises and for the roomy arrangement of the various machines necessary in the treatment of scoliosis and static deformities, and a massage room. The ideal clinic should also contain a swimming bath and a gymnasium, together with a hydrotherapeutic department. Where such a department already exists, it is usually easy to arrange for patients from the orthopædic department to use it on certain fixed days. Here, as in the other units, a certain amount of overlapping is unavoidable, but efficient administration and management will readily overcome any difficulties that may arise and secure that close co-operation between the various departments which is the essence of success in a modern up-to-date hospital.

Fig. 1.—A Corner of the Zander Room, Royal National Orthopædic Hospital.

Ideal treatment departments exist in several institutions abroad. Of these, the Widener Home at Philadelphia is undoubtedly one of the most interesting and complete. The newer "Victor Lenel Institute" in the Neckarthal, although not strictly speaking an orthopædic hospital, has an interesting swimming bath of the type which we should like to see in every orthopædic department. A photograph and description of it appears in this year's Report of the Medical Officer of Health for Mannheim. Institutes with excellent treatment

* Previous articles appeared on November 1, 15, 29 and December 13.

departments exist at Bologna (Instituto Orthopedico Rissoli), at Moscow (Children's Hospital), at Berck-sur-Mer, Turin, Bonn, Berlin, Copenhagen, Munich, and in several American cities. In this country, unfortunately, with the possible exception of the Royal National Orthopædic Hospital in Great Portland Street, no hospital can be said to have an adequate and really efficient treatment division attached to an orthopædic department.

The Exercise Room.

This should be a roomy, well-ventilated, and carefully heated apartment. The best flooring here is parquet, or linoleum laid on cement, although, if the floor is used in crawling exercises, the linoleum is apt to become unsightly very soon, whereas the parquet wears excellently, and, in fact, seems to improve with the polish it derives from the patients' knees and elbows. We have given a description of the crawl method of treatment in cases of structural scoliosis (see The Hospital, July 20, 1912, p. 417) and there is no need to explain the method in detail; suffice it to say, that the patients—usually, of course, children—are made to crawl on all fours under the supervision of a nurse or assistant. For this a smooth floor, free from splinters, is absolutely essential; it must also be fairly warm (hence terrazzo will do). Practically no other apparatus is required; all the patients need are guards of leather or thick canvas for their knees, to prevent undue chafing, and fairly thick woollen or leather gloves. But for the treatment of scoliosis other apparatus is required if other methods, in many cases very useful where the Klapp crawl method cannot be used, are to be employed. And as the exercise room will be chiefly devoted to scoliotic cases, it is necessary to pay some attention to the various types of apparatus needed. The annexed photograph shows the apparatus used in the National Orthopædic Hospital.

In German hospitals the apparatus room (Pendelsaal) is usually adequately furnished. It has a firm parquet flooring, affording a good base for the various machines, and has a good window light admitting direct sunlight during the hours when the machines are in use—generally between ten in the morning and four in the afternoon. The furnishing of the exercise room is a matter largely of individual choice; different surgeons prefer different kinds of apparatus. The consensus, however, is largely in favour of the Zander machines. Many clinics prefer the Kruckenberg apparatus, of which the essential types are the treadle and resistance apparatus for flexion and extension of the knee joint, the "Universal resistance" apparatus for use in upper extremity lesions, and a somewhat similar machine for the lower extremity; the prices for these vary from £12 to £24 each. Modifications and improvements of these machines are constantly catalogued, and to be up to date the orthopædist should keep in touch with newer inventions, and acquire those that promise a real improvement. Among these are the shoulder fixation apparatus of Wullstein, and the Gocht instruments for hip flexion and extension. A much more expensive combination is found in the Becker machines, in which provision is made for the employment of hot air. Additional machines are those for extra movements such as body flexion, as in the Knoke-Dressler type, the Trennert, Rossel-Schwartz, and Staffel machines for employment in ankylosis cases after forcible reduction, and a variety of other types. We may mention here, just to give the reader some idea of the variety of machines recently introduced, the following types which have all been lauded by the German school. Caro's simplified apparatus, which takes up little room, and is relatively cheap. Heerman's lever and swing machines; Hohmann's improvised apparatus; Baehr's finger machine, and the more recent modifications of the older Zander and Schultess machines. Indispensable apparatus in

Fig. 2.—The Massage Room, Royal National Orthopædic Hospital, London.

the manipulative treatment of scoliosis are the Barwell-Hoffa frame, the inclined plane with head extension, the extension frame for plaster work, Lubinus' modification of the Hoffa frame, Wullstein's correction apparatus, and Beely's forcible correction frame. In addition there are needed low tables or couches, plain and with leather padding; these should be similar to the massage benches used in the massage room. Ordinary Swedish gymnastic apparatus should also be provided, and, indeed, the furnishing of the exercise room can be extended indefinitely, the choice being limited by the expense alone. As a minimum, however, it may be taken that such a room should contain at least three machines of the Zander or Kruckenberg type, providing for flexion and extension of all the lower and upper limbs, apparatus for the treatment of scoliosis, including extension and correction apparatus, slings and suspenders for fixing plaster corsets, and some simple gymnastic apparatus. The cost of this equipment can hardly be estimated below £150.

Provision must also be made for the application of the newer methods of treatment by hot air or by Bier's apparatus. The additional cost entailed by providing this apparatus is not very great, and the advantages justify the expense.

The massage room is an indispensable adjunct to the exercise room, but its furnishing costs much less, although here again there is a wide range of choice, especially if machinery is to be extensively employed. Many orthopædists, however, prefer to trust to skilled assistants rather than to machinery. The latter does the work very well, under skilled supervision, but the apparatus is expensive, and often gets out of order.

## Figures and Tables

**Fig. 1. f1:**
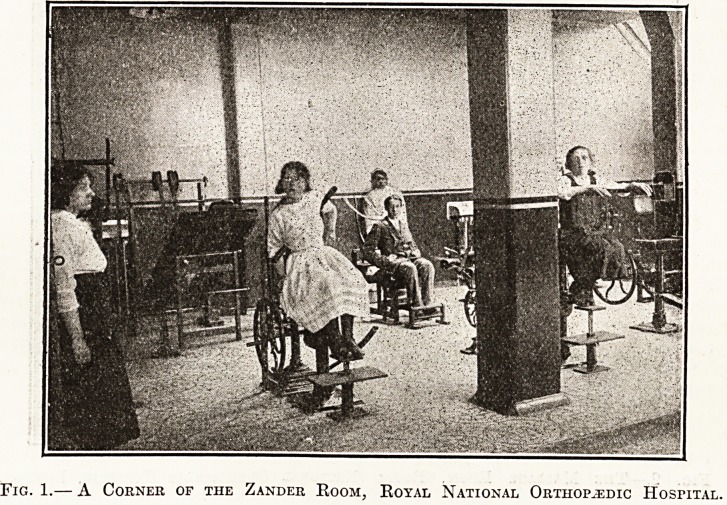


**Fig. 2. f2:**